# A Soft Transporter Robot Fueled by Light

**DOI:** 10.1002/advs.201902842

**Published:** 2020-01-20

**Authors:** Marina Pilz da Cunha, Sebastiaan Ambergen, Michael G. Debije, Erik F. G. A. Homburg, Jaap M. J. den Toonder, Albert P. H. J. Schenning

**Affiliations:** ^1^ Institute for Complex Molecular Systems Eindhoven University of Technology Den Dolech 2 5600 MB Eindhoven The Netherlands; ^2^ Laboratory of Stimuli‐Responsive Functional Materials & Devices Department of Chemical Engineering and Chemistry Eindhoven University of Technology P.O. Box 513 5600 MB Eindhoven The Netherlands; ^3^ Department of Mechanical Engineering Eindhoven University of Technology P.O. Box 513 5600 MB Eindhoven The Netherlands

**Keywords:** light‐driven soft robots, liquid crystal soft robots, photoactuators, untethered soft robotics

## Abstract

Mobile organisms with ability for locomotion and transportation, such as humans and other animals, utilize orchestrated actuation to perform actions. Mimicking these functionalities in synthetic, light‐responsive untethered soft‐bodied devices remains a challenge. Inspired by multitasking and mobile biological systems, an untethered soft transporter robot with controlled multidirectional locomotion with the ability of picking up, transporting, and delivering cargo driven entirely by blue light is created. The soft robot design is an ensemble of light‐responsive liquid crystalline polymers that can harness motion either collectively or individually to obtain a high degree of motion control for the execution of advanced tasks in a dry environment. Through orchestrated motion of the device's “legs”, single displacement strides, which exceed 4 mm and can be taken in any direction, allow for locomotion around objects. Untethered cargo transportation is demonstrated by a pickup and release mechanism using the device's “arms”. This strategy demonstrates the constructive harnessing of orchestrated motion in assemblies of established actuators, performing complex functions, mimicking constructive behavior seen in nature.

## Graphical abstract

Soft robotic devices strive to mimic the multidirectional locomotion and transportation performed by living creatures. Here, an untethered polymer device is shown to achieve macroscopic functions including grasping, carrying, walking, and releasing of objects performed and controlled solely with light. The functions are completed through constructive orchestrated motion of multiple liquid crystalline actuators that compose the soft robotic device.

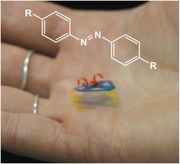

## 1 Introduction

Multidirectional locomotion and transportation are crucial functions for humans and other animals. Orchestrated movement of individually addressed limbs allows for the generation of constructive macroscopic motion and the execution of useful actions. Scientific fascination for nature's machineries has fueled an expanding field of research focused on mimicking these essential functions of natural systems into synthetic soft robots with untethered control.^1^, ^2^, ^3^, ^4^, ^5^, ^6^ Responsive materials^7^, ^8^, ^9^, ^10^, ^11^ with actuation triggered by heat, light, and humidity are of special interest for soft robotics to eliminate the need for wiring or tubing connectivity between the device and the external controllers, allowing for lighter, autonomous, and more versatile soft robots.^1^, ^12^ Current developments in soft microrobotics aspire toward systems with locomotive freedom as well as the ability to perform useful tasks, such as cargo handling.^13^, ^14^ Presently, small‐scale devices with remotely controlled locomotion and transportation abilities are driven by magnetic fields which require extensive external setups.^14^, ^15^ Light as an untethered stimulus allows for greater versatility as well as high degree of control and temporal resolution.^2^, ^6^ The current state of the art of light‐driven robots focuses on devices based on single actuators and currently no system has demonstrated both locomotive freedom and transportation ability. To generate macroscopic functions including grasping, carrying, walking, and releasing of objects performed by living creatures requires integrated actuator assemblies working in concert, and cannot be achieved by single light‐driven actuators working alone.

Liquid crystalline networks (LCNs) have been successfully employed to remotely and wirelessly achieve controlled bending,^16^, ^17^ rolling,^18^ twisting,^19^, ^20^ and oscillating motions,^21^, ^22^, ^23^ appearing as promising candidates for the mobile components in untethered soft robots in dry environments.^24^ Light response is popularly employed in LCN‐based soft robots and is achieved through the inclusion of photoactive molecules into the polymer network in which the molecular architectures dictate the nature of the movement.^25^, ^26^ Azobenzene chromophores are often utilized as the photoswitching molecules due to their compatibility with the liquid crystal (LC) network, as preservation of molecular alignments is essential for actuation.^27^ In recent years, a variety of centimeter‐sized light‐driven soft robots based on LCNs have been demonstrated,^6^ yet design and locomotion freedoms in dry environment remain limited to one dimension, either forward^28^, ^29^, ^30^ or forward and backward.^31^, ^32^, ^33^, ^34^, ^35^, ^36^ Unidirectional locomotion strategies such as inchworm,^28^, ^37^ snail,^34^ and caterpillar‐like^33^, ^35^ movements have been explored. While light‐activated LCN grippers have been explored,^38^, ^39^ transportation of cargo by light‐driven walking devices has not yet been demonstrated. In this work, we construct the first light‐driven soft robot capable of untethered multitasking through the integration of multiple actuators and two different azobenzene derivatives. The soft robot is driven by blue light and can walk around objects, with controlled multidirectional locomotion. Additionally, it performs multiple complex movements enabling pickup, grasping, transportation, and directed deposition of cargo.

## 2 Results

### 2.1 Design of Light‐Fueled Soft Robot

**Figure 1:**
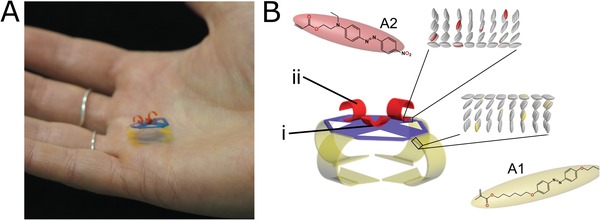
The soft robot is composed of four identical light‐responsive liquid crystalline polymer films that operate as legs, connected to and supporting a polymeric hub (polypropylene) which hosts an assembly of liquid crystalline films acting as soft robotic arms that operate as the device's cargo holder and handler. The hub is symmetric with four triangular “cuts” for decreased weight while maintaining the supportive structure. A) The soft robotic device is of centimeter size (spanning 2 cm across) and weighs 20 mg. B) A schematic depiction of the device, demonstrating the two different light‐responsive azobenzene chromophores (depicted in yellow and red) used to separately address the legs (yellow) and arms (red) of the cargo handler.

The soft robot design is an assembly of curled liquid crystal polymer films, serving as the active device components, and a lightweight static polypropylene polymeric hub which acts as a connective hub for all responsive segments of the device, **Figure** **1**A. The integrated robot consists of four LCN “legs” containing the yellow colored photoswitch A1 in Figure 1B, two LCN “arms” ((ii) in Figure 1B) containing the red colored photoswitch A2, and a small LCN gripper ((i) in Figure 1B) composed of two LCN films (photoswitch A2) placed orthogonally to each other. This centimeter‐sized soft robot can be remotely controlled to perform multidirectional locomotion (using the yellow LCN legs in Figure 1) and collection, holding, and deposition of cargo (by means of the red LCN arms on top of the static hub, Figure 1). Actuation of the device is achieved through illumination via a collimated light‐emitting diode (LED) source at wavelengths absorbed by the photoswitch. To achieve selective addressability, the polymer films realizing locomotion and cargo handling contain different photoactive azobenzene derivatives, each with different sensitivities to the excitation light. Both legs and arms utilize the curled shape of the LCN relaxed state, with the 12 mm long legs neatly curling under the static hub of the device in nonilluminated conditions. The four‐legged design allows the soft robot to have directional freedom of locomotion around objects (Movie S1, Supporting Information). All four LCN legs are designed with a 1:3 (width:length) aspect ratio with a broader base (10 mm width), devised to improve frictional interaction between the robot leg and surface (vide infra). The cargo handler arms are composed of two parts: a gripper constructed by two orthogonal LCN films (5 mm in length and 2 mm in width) placed on the center of the static hub (labeled (i) in Figure 1B) acting to secure the cargo during transportation and two LC films (8 mm in length and 2 mm in width), placed at 90° to each other ((ii) in Figure 1B) to act as the device's arms, enabling both actuation for cargo pickup (Movie S2, Supporting Information) as well as cargo off‐loading (Movie S3, Supporting Information).

### 2.2 Light‐Triggered Actuation of the Liquid Crystal Networks

**Figure 2:**
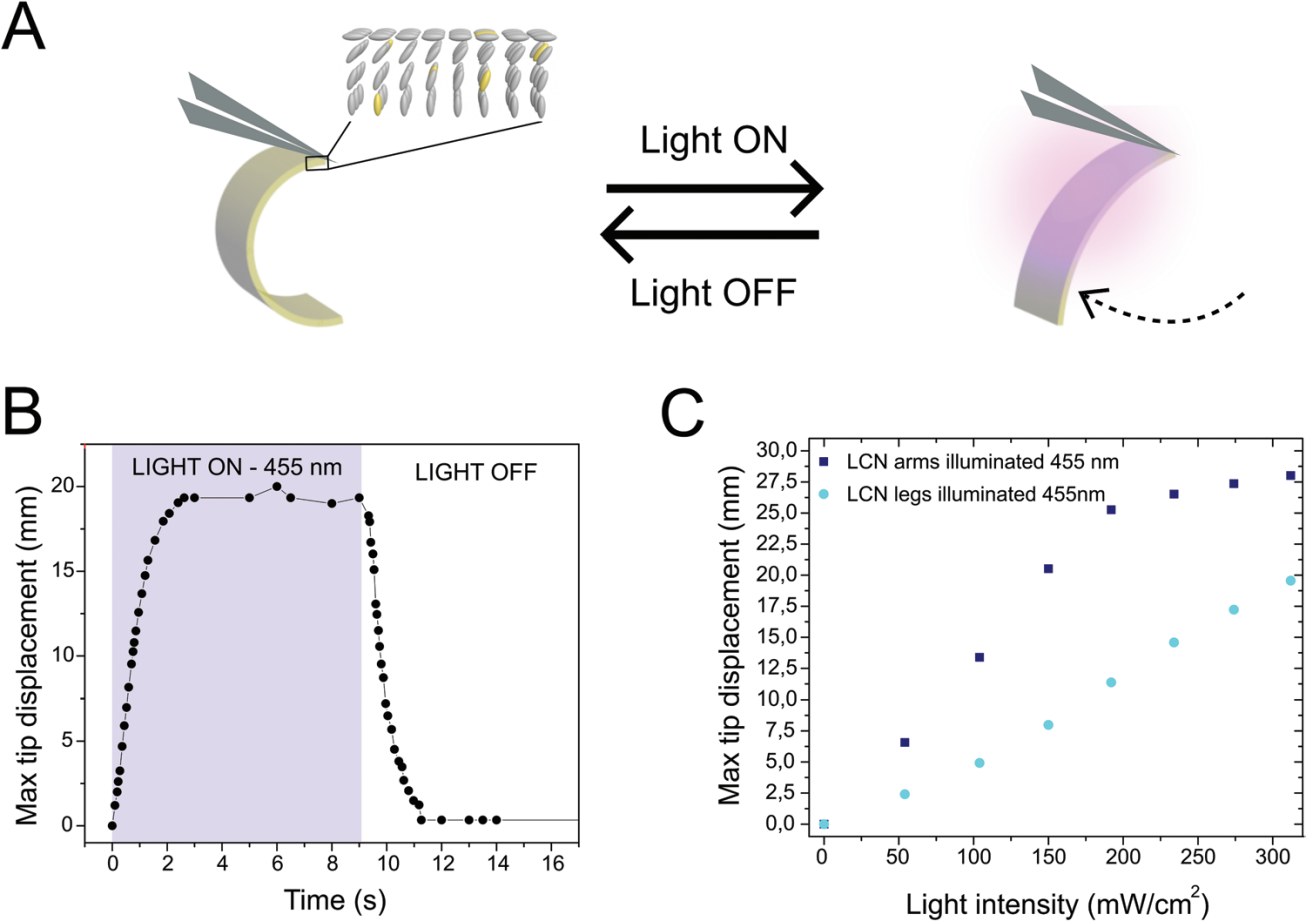
Characterization of a photoresponsive liquid crystal network composed of photothermal azobenzene chromophores. A) Illustration of the actuation of a splay aligned network, secured on one side by tweezers. The LCN consists of liquid crystals (gray) and photoisomers (yellow) responding to illumination. Starting from a curled geometry, the film unbends toward its planar surface when illuminated. B) Plot displaying the high temporal resolution of the unbending and recurling processes of a LCN strip (2 cm in length and 0.4 cm in width), containing photoswitch A1 corresponding to application (“light on”) and removal (“light off”) of illumination, respectively. C) Graph depicting the actuation performances of the LCN arms and legs as a function of illumination intensity.

The LCN films used in the robot are fabricated through the photopolymerization of a LC monoacrylate (40.5 mol%) and a diacrylate (56.5 mol%) mesogen and 2 mol% of azobenzene chromophore, Figure S1 (Supporting Information). Photopolymerization is carried out within a 20 µm gap glass cell containing polyimide layers with homeotropic alignment at one surface and planar alignment at the opposing side to realize a splay alignment through the network's thickness; see the Experimental Section for fabrication details. The initial curled state of the LCN films is a result of the elevated temperature during polymerization, causing anisotropic volume shrinkage upon cooling to room temperature. The precurl is essential for the locomotion mechanism of the device (vide infra). By using photothermal monoacrylate azobenzene derivatives, actuation arises when the photoswitch absorbs specific wavelengths of light (Figure S2, Supporting Information) and releases heat as a result of the isomerization processes (Figure S3, Supporting Information).^37^ The released heat triggers a macroscopic response originating from the anisotropy in the thermal expansion of the splay network, Figure S4 (Supporting Information). Employing monoacrylate azobenzene photoswitches allows for reversible and rapid (seconds) actuation. Light‐triggered macroscopic actuation of the LCNs manifests itself as film unbending, from an initial curled state, to a straighter film, **Figure** **2**A;^40^ the uncurling deformation is independent of the incident illumination direction and no signs of actuation fatigue are present over dozens of illumination cycles.

The large unbending deformations triggered by light exposure have a high degree of temporal resolution. Forward (unbending) and backward (recurling) actuations are initiated directly by the exposure or removal of light, respectively, Figure 2B. The rapid second‐scale response of the LCNs contributes toward increased speed of operation of the soft robot. The extent of actuation of the LCN can be directly controlled by the intensity of the light stimulus: higher intensities result in larger deformation amplitudes, Figure 2C. LCNs doped with the red azobenzene derivative (A2) used for the device's arms display a more efficient actuation profile, with lower intensities resulting in far larger amplitudes when compared to the yellow azobenzene (A1) molecule‐doped legs. This actuation efficiency difference is a result of the red chromophore's higher extinction coefficient (Figure S2, Supporting Information) and allows for selective addressability of the LCN components.

### 2.3 Light‐Driven Locomotion

**Figure 3:**
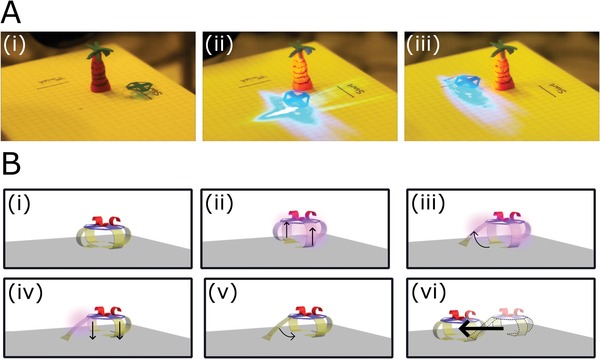
Light‐driven locomotion of the soft robot in multiple directions over a paper surface. A) The simple robot (without integration of LCN arms) is demonstrated to move from its initial position (i) around a palm tree (ii) toward its finish line (iii). B) The walking mechanism for one stride consists of three main steps and two light sources.

First, we focus on the characterization of the walking behavior of the soft robot. Through remotely controlled light‐triggered actuation of the LCN legs, our soft robot performs a walking‐like motion. The symmetric distribution of the device's identical legs allows for freedom in locomotion, with the unprecedented ability to not only walk in forward and reverse directions, but also around objects, **Figure** **3**A. An illumination sequence of three main steps, using two collimated light sources, allows the robot to perform a single stride covering a distance that exceeds 4 mm, Figure 3B. The first step in initiating locomotion is the liberation of the “striding leg” from under the hub, achieved through low intensity side illumination (455 nm at 150 mW cm^−2^) of the two legs located orthogonal to the “striding leg,” which slightly lifts the device, step (ii) in Figure 3B. Note that only a single LED source is necessary to cause the lifting motion. The low absorbance of blue light by the yellow‐azobenzene legs eliminates the necessity of addressing each “lifting leg” individually as a significant intensity of light incident on one leg penetrates to also illuminate the distal LCN leg. Addressing the striding leg with the other light source (455 nm, at an ≈45° angle to the surface), initiates an unbending motion, (iii) in Figure 3B. Removal of the illumination of the side legs then lowers of the robot, bringing the striding leg again into contact with the surface, (iv) in Figure 3B. Ceasing illumination of the striding leg initiates the forward stride, fueled by the elastic force which returns the striding leg into its bent position under the hub, (v) in Figure 3B. As a result, the striding leg effectively drags the device forward, (vi) in Figure 3B. As any leg can act as the “striding leg” or “lifting leg,” the device is able to follow a zigzag walking pattern to reach a destination in any direction.

#### 2.3.1 Characterization of the Light‐Driven Locomotion

**Figure 4:**
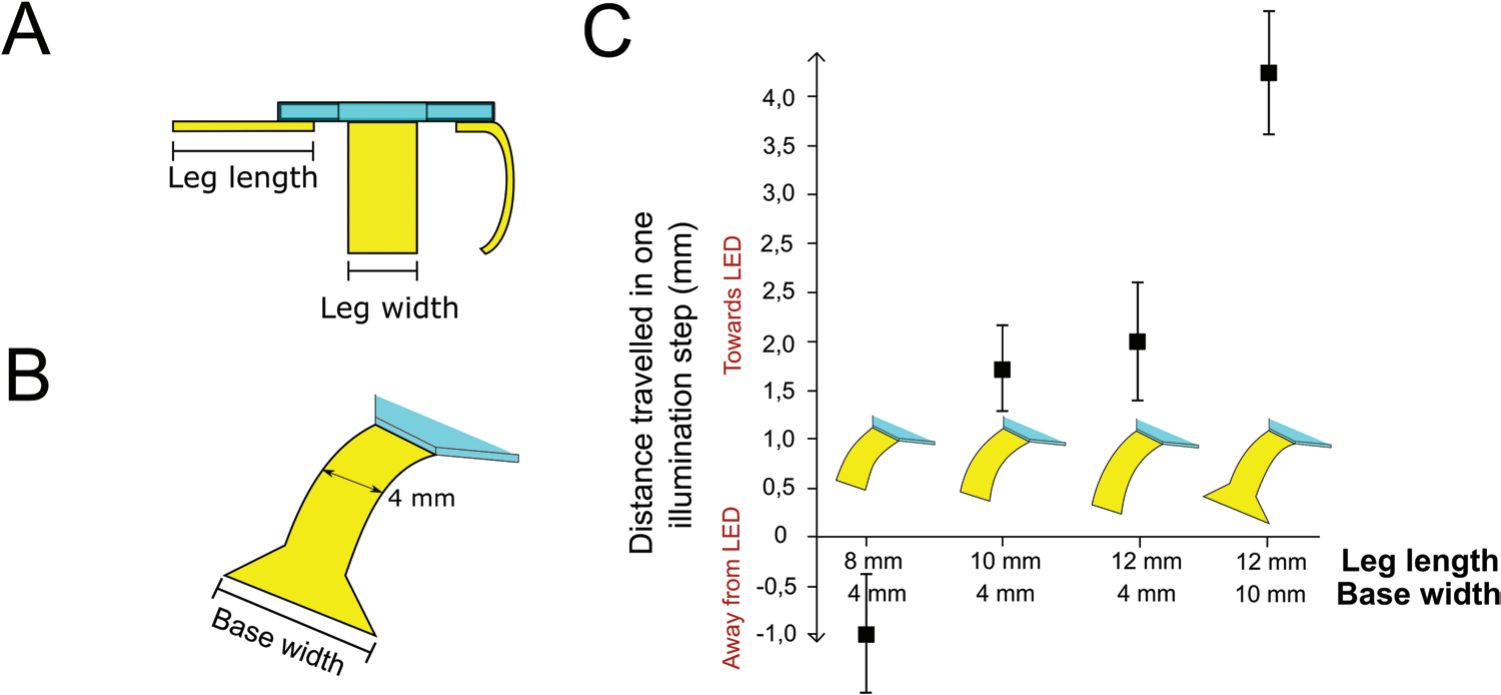
Investigation of the importance of leg dimension and shape for locomotion. A) Schematic depiction of the robot, showing leg length and width dimensions. B) Schematic drawing of the leg design with a wider leg base which enhances walking locomotion. C) The effect of leg length and width on both the directionality and efficiency of locomotion. We plot the average distance covered by one illumination cycle (described in Figure 3B) for robots with varying leg dimensions and the standard deviation of these values for ≈10 steps. We observe that short 8 mm legs result in walking in a direction away from the illumination and longer, 10 and 12 mm legs, in locomotion toward the light source. Little variation is observed between legs of 10 and 12 mm, yet an increase in leg base largely increases the distance covered by one stride.

To illustrate the crucial role of our design in the robot's ability for multidirectional locomotion, we demonstrate the impact of design choices on the locomotion. Of particular interest are the effects on the distance covered by a single stride following alterations in leg dimensions, considering both leg length and shape, **Figure** **4**A,B. We systematically investigated the effect of leg length (8, 10, or 12 mm) on the light‐driven walking motion of the device. Surprisingly, we found that the directionality of locomotion changes with alterations in the leg length, with short 8 mm legs showing locomotion away from the source of illumination and longer legs' locomotion toward the light, Figure 4C. In longer leg designs, the LCN legs curl under the hub when not illuminated. The short 8 mm (4 mm wide) legs are not long enough to completely curl: the leg edges are instead in direct contact with the surface (Figure 4A). The short legs are inefficient in the initial lifting motion (step (ii) in Figure 3B), failing to properly liberate the striding leg. A single high intensity illumination directed at any leg (250 mW cm^−2^) results in a small thrust motion, displacing the device away from the light source.

Considering that the walking motion resultant from the longer (12 mm) LCN legs displayed the most consistent and efficient locomotion, we investigated how to further improve the design to increase the distance covered by one stride. Since the driving force for displacement is in the elastic recovery of the strider leg to its nonilluminated curled shape while being in contact with the surface, which results in traction that pulls the robot forward, we explored the possibility of increasing the traction between the strider leg and the surface during the recurling process. By changing the geometry of the leg's end (Figure 4B), we minimally impact the bending magnitude of the film in its nonilluminated state (by keeping the leg aspect ratio constant) but increase the LCN film edge in contact with the paper surface during forward pulling motion (step (v) in Figure 3B). The increase in surface area greatly enhances the traction and decreases foot slippage at the interface between the LCN leg and the surface, significantly increasing the overall distance covered by one stride, Figure 4C. The striding step is the determining action in the locomotion mechanism for our robot and the striding motion is controlled by the capacity of the LC network to elastically recover its initial curl. With a 4 mm displacement per actuation cycle and one actuation cycle under 8 s, we estimate the speed of our soft robot to be 0.5 mm s^−1^. Additionally, we observed that the substrate surface has significant impact on the degree of locomotion. When exchanging the paper surface for either a low (glass) or high (sandpaper) friction surface, no locomotion is observed. In both the cases, the robot fails to complete a striding step either through foot slippage of the strider leg (on glass surfaces), or the inability for the traction on the strider leg to overcome the increased friction on the other legs curled under the hub (for sandpaper surface), leaving the robot in the position depicted by step (v) in Figure 3. These observations demonstrate that the surface interactions between the LCN films and the substrate surface are fundamental for the locomotion mechanism. We suggest that for effective locomotion to happen, a compromise should exist between enough traction generation for the strider leg recurling to cause a net displacement during forward stride, and sufficient slippage at the other, curled, legs to allow this traction to pull the robot forward. Additionally, inhomogeneity of the substrate surface can also hinder uncurling of the striding leg from under the robot and hence impede locomotion.

### 2.4 Light‐Driven Pickup, Transportation, and Release of Cargo

**Figure 5:**
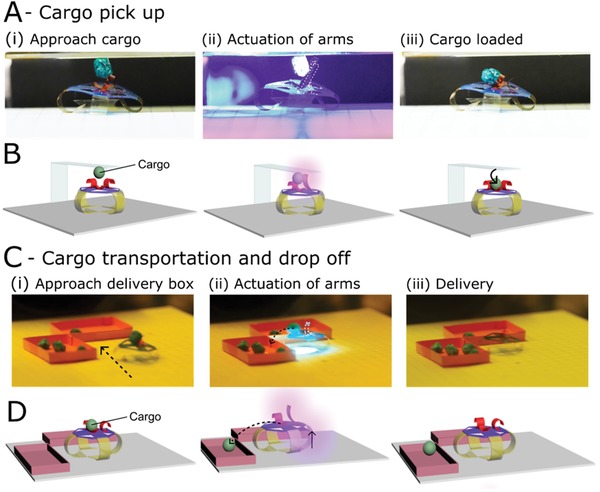
Cargo pickup, transportation, and release. A) Snapshots of the untethered cargo pickup activity. B) Schematic depiction of the pickup mechanism. The cargo hangs from a glass surface coated with an adhering coating to keep the cargo in place until the robot's arms actuate and release it. C) Snapshots of the untethered transportation and cargo release. D) Schematic depiction of the release mechanism when the robot approaches the delivery boxes. For cargo release, both the LC gripper (located in the center of the hub) and one of the “arms,” are actuated to cause gripper opening and thrust the cargo in either one of the boxes. Additionally, actuation of one LC leg causes the robot to slightly lift, enhancing the directed cargo thrust into the delivery box.

Finally, we show the novel ability of our device to perform useful functions such as remotely controlled pickup of a load, its transportation, and release at a specific location. Our demonstration is the untethered pickup of a soft styrofoam cargo (5 mg) which hangs from a tacky glass surface (**Figure** **5**A) and its transport and release into a delivery box (Figure 5C). Through remotely controlled locomotion, the robot is moved in position under the cargo while light‐driven actuation of the LCN arms causes the films to uncurl. In the actuation process, the arms encounter the hanging cargo, causing it to fall into the LCN gripper, Figure 5B. In this actuation step, a wide focus light is employed (at an intensity of 200 mW cm^−2^) so as to simultaneously activate all segments ((i) and (ii) in Figure 1B) of the cargo handler. The wide focus allows light to also reach the device's legs, yet the lower sensitivity of the chromophore in these active sites (A1) does not cause any “striding” actuation from any legs. The difference in optical absorbance between the azobenzene films used in the legs and arms eliminates the need of finely focused light sources such as laser beams to enable specific actuators in the device, even though the actuators are in close proximity. Removal of illumination results in the recurl of the LCN arms and closing of the central gripper, securing the cargo so that the robot can transport the load without dropping it.

Upon reaching a destination, the cargo can be offloaded, Figure 5C. Illumination of the cargo gripper and one of the LCN arms allows for the gripper to open, and one of the arms to thrust the cargo from the hub into the delivery site. The presence of two arms allows for an expanded choice of delivery as the cargo can be easily off‐loaded in two directions. To aid the cargo delivery, a low‐intensity illumination of one LCN leg lifts the robot asymmetrically, creating a slope for the cargo to descend, Figure 5D. It should be noted that when the robot is excessively loaded, locomotion is dramatically hindered as not only is the lifting activity of the side legs (step (ii) in Figure 3B) deterred by the extra force needed to lifted the weighed hub, but also, once the striding leg is activated, the recovery of the film's curvature is impeded (step (v) in Figure 3B). The loading capacity limitation is directly correlated to the thickness of the LCN legs. In turn, film thickness directly influences the degree of molecular organization through the network which controls the prebend curvature of the legs. As the prebend curvature is essential for the locomotion strategy, the direct balance between molecular organization and mechanical performance sets restrictions in the design of leg dimensions. From experiments, we have found that inhibition of locomotion is present at a loading of 50% the robot's own mass, limiting transportation ability of the soft robot to lighter loads. Additionally, we show that the robot is also able to “guide” cargo that is located on the surface. This cargo transportation is done by effectively pushing the load over the surface during liberation of the striding leg during locomotion ((iii) in Figure 3B), a motion that resembles a kick, Movie S4 (Supporting Information). This motion can be utilized for cleaning or removing objects from a surface.

## 3 Conclusion

The uniqueness of our untethered soft robot is demonstrated in the harnessing of constructive motion from an assembly of well‐established actuators to achieve versatile mobility and transportation function. The concerted orchestration of several actuators in a single device assimilates the way organisms utilize individual limbs for constructive function. Motion control is attained exclusively by light control, overcoming present challenges in the field of soft microrobotics such as removal of control systems from the individual robot and eliminating the need for direct tethering, as is the case for electrically driven systems.^41^ To the best of our knowledge, we present the first fully light‐driven soft transporter robot with controlled multidirectional locomotion and ability to pickup, transport, and deliver cargo. The design allows for control of different segments in the device and full freedom in locomotion, allowing the device unprecedented versatility of motion around objects. Our study sets a step forward in the engineering of orchestrated actuation in assemblies of light‐responsive actuators, enabling the completion of functional tasks that cannot be performed by single actuators alone.

## 4 Experimental Section

*Fabrication of Liquid Crystalline Network*: The liquid crystal polymer network was produced from two liquid crystalline monomers: a monoacrylate, 2, (RM 23; 40.5 mol%, Merck) and a diacrylate, 1, (RM 82; 56.5 mol%, Merck), initiated by a photoinitiator (Irgacure 819, 1 mol%, Ciba), Figure S1 (Supporting Information). Light responsivity was achieved with the addition of a commercially available azobenzene chromophore (2 mol%) with a fast *cis*–*trans* isomerization, A2, (DR1A, Sigma‐Aldrich) or with azobenzene derivative A1, having a longer *cis* lifetime (Synthon). Prior to polymerization, the monomers were dissolved in dichloromethane to obtain a homogeneous mixture; the solvent was subsequently evaporated. Custom‐made cells were prepared by gluing together two glass slides coated with differing polyimide alignment layers (for a splay alignment; one slide with planar and the other with homeotropic alignment layers (Optimer AL 1051 (JSR Micro) and 5661 polyimide (Sunever), respectively)). Glass bead spacers (20 µm diameter) were incorporated into the glue to achieve controlled cell thickness. The cells were filled at 95 °C, at which the LC mixture was isotropic through capillary action. Subsequently, the filled cell was cooled to 80 °C, at which temperature the LC mixture was nematic. Photopolymerization of the reactive mesogens was done at 80 °C with an Exfo Omnicure S2000 lamp; subsequent thermal treatment at 120 °C for 10 min released thermal stresses arising from polymer shrinkage during polymerization. After polymerization, the cell was opened, and the films were peeled from the glass with razor blades and cut into the required shapes using the same blade.

*Soft Robot Dimensions*: The two orthogonally placed LCN films acting as cargo holder measured 1.5 mm by 6 mm in width and length, respectively. The films acting as “arms” measured 1.5 and 10 mm in width and length, respectively. The polymer hub was cut from a 0.12 mm thick polypropylene sheet. The hub was an octagon, with dimensions shown in Figure S5 (Supporting Information). The hub had four identical cuts in the center so as to reduce the hub weight. The LCN films were attached to the polymeric hub with thin strips of double‐sided tape.

*Actuation of Soft Robot*: The soft robot was actuated while placed on a paper surface, Figure S6 (Supporting Information). Illumination was performed with a LED light source emitting 455 nm (Thorlabs M455L3‐C2) mounted with a collimator (ThorLabs SM2F32‐1) and driven by a controller (also ThorLabs). The distance between the LED source and the focus was ≈10 cm.

## Conflict of Interest

The authors declare no conflict of interest.

## Supporting information

Supporting Information

Supplemental Video 1

Supplemental Video 2

Supplemental Video 3

Supplemental Video 4
