# Supplementary material for: A Soft Transporter Robot Fueled by Light
Source: Adv Sci (Weinh). 2020 Jan 20;7(5):1902842. doi: 10.1002/advs.201902842 (PMC7055549; doi:10.1002/advs.201902842)
Supplement: Supplementary file 1 — Supporting Information [file ADVS-7-1902842-s001.pdf]

Copyright WILEY-VCH Verlag GmbH & Co. KGaA, 69469 Weinheim, Germany, 2020.

ADVANCED SCIENCE
Open Access

Supporting Information

for *Adv. Sci.,* DOI: 10.1002/advs.201902842

A Soft Transporter Robot Fueled by Light

Marina Pilz da Cunha, Sebastiaan Ambergen, Michael G. Debije, Erik F. G. A. Homburg, Jaap M. J. den Toonder, and Albert P. H. J. Schenning**

Copyright WILEY-VCH Verlag GmbH & Co. KGaA, 69469 Weinheim, Germany, 2020.

Supporting Information

A soft transporter robot fueled by light

Marina Pilz da Cunha, Sebastiaan Ambergen, Michael G. Debije, Erik F. G. A. Homburg, Jaap M. J. den Toonder, Albert P. H. J. Schenning**

1
2
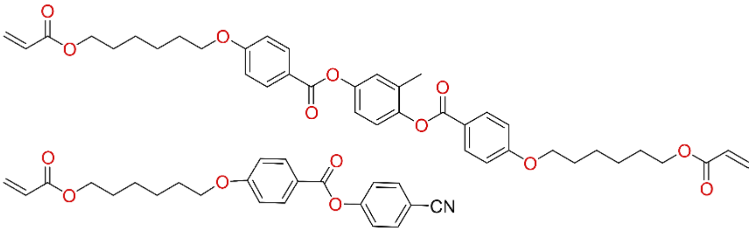

Figure S1
Molecular structures of LC mesogens diacrylate (**1**) and monoacrylate (**2**). The LC mixture contains of 56,5 mol% of the diacrylate and 40,5 mol% of the monoacrylate LC.

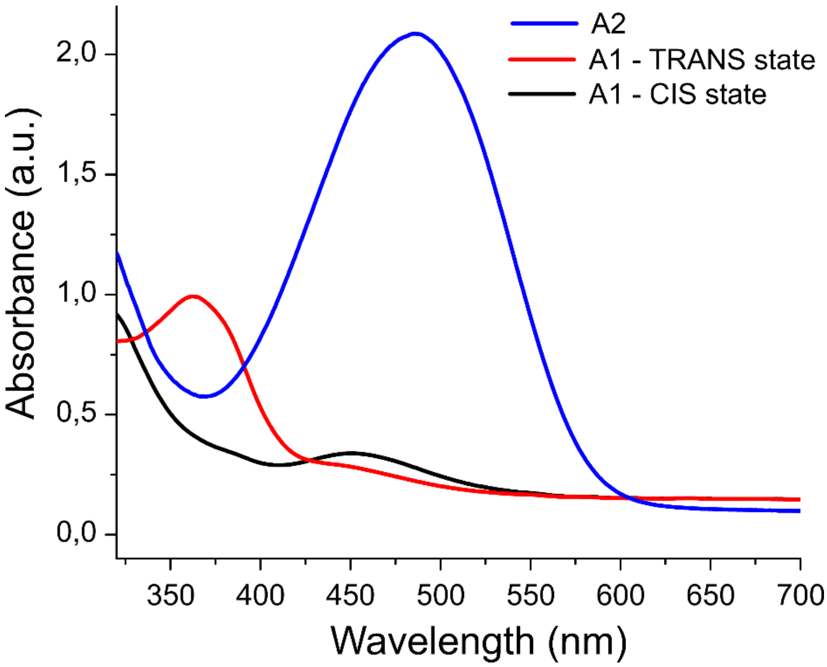

Figure S2
Absorption spectra of the two splay networks containing 2 mol% of either azo derivative **A1** or **A2**. Azobenzene derivative **A2** has a very short *cis* lifetime, in the millisecond range, hence the single absorbance spectrum. Azobenzene derivative **A1** has a longer *cis* state lifetime. The *cis* state spectrum is measured after film exposure to 365 nm light for 30 seconds.

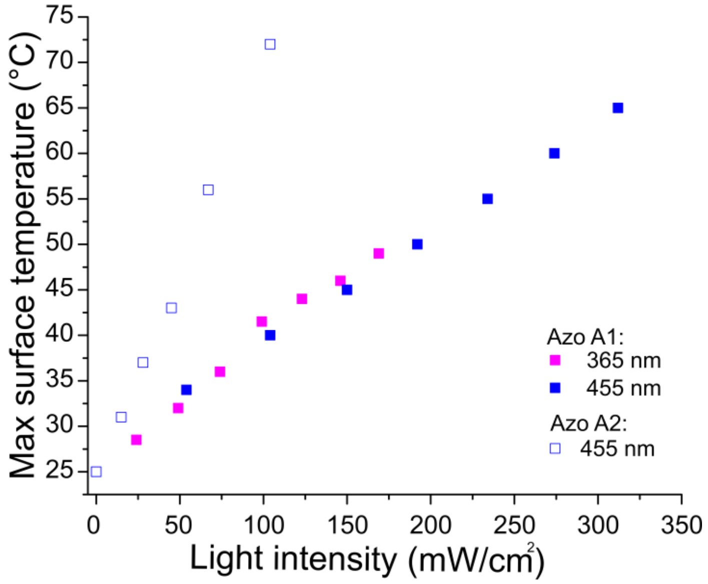

Figure S3

Surface temperature profile of the LCN films (doped with either azo A1 or A2) upon illumination with 365 or 455 nm LED source.

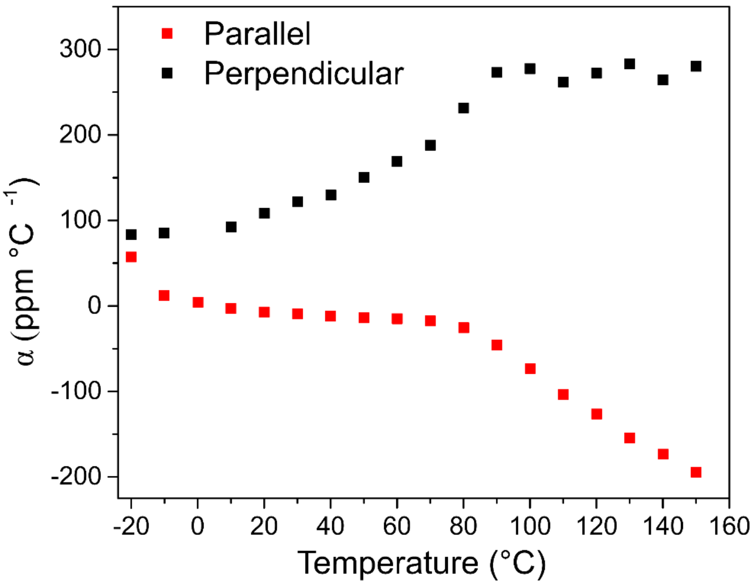

Figure S4

Coefficients of thermal expansion (CTE) of the LC networks parallel (red squares) and perpendicular (black squares) to the molecular long axis, respectively. The reference temperature is -20 °C.

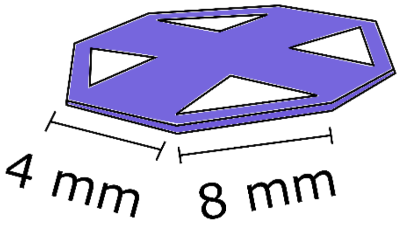

Figure S5
Schematic depiction of the polypropylene polymeric hub with dimensions.

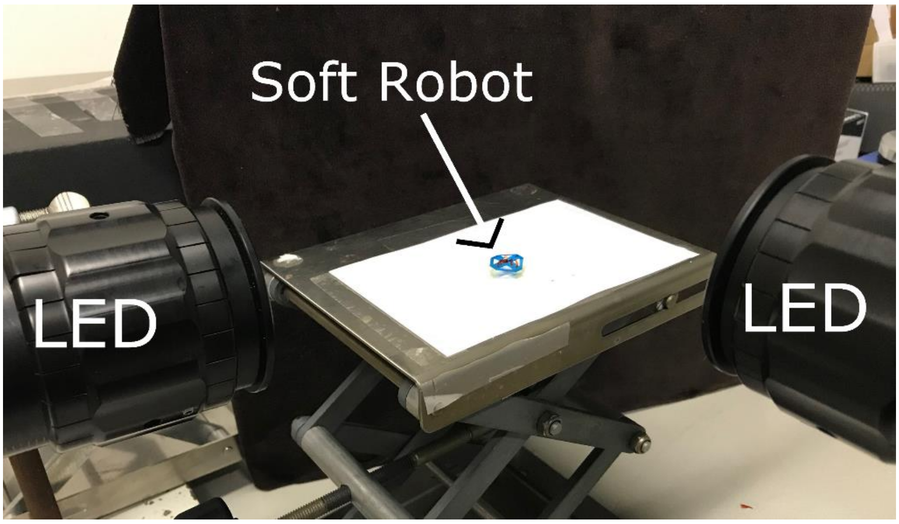

Figure S6
Picture of the setup used to actuate the soft robot. For locomotion two collimated light sources are used. The robot moves over a paper surface.
